# Combined Effects of Microgravity and Chronic Low-Dose Gamma Radiation on *Brassica rapa* Microgreens

**DOI:** 10.3390/plants14010064

**Published:** 2024-12-28

**Authors:** Sara De Francesco, Isabel Le Disquet, Veronica Pereda-Loth, Lenka Tisseyre, Stefania De Pascale, Chiara Amitrano, Eugénie Carnero Diaz, Veronica De Micco

**Affiliations:** 1 Department of Agricultural Sciences, University of Naples Federico II, 80055 Portici, Italy; sara.defrancesco@unina.it (S.D.F.); depascal@unina.it (S.D.P.); 2 Institute of Systematic, Evolution, Biodiversity of Sorbonne University, 75005 Paris, France; isabel.le-disquet@mnhn.fr; 3 Evolsan-GSBMS, Faculté de Santé, University of Toulouse III, 31062 Toulouse, France; veronica.pereda@univ-tlse3.fr (V.P.-L.); lenka.tisseyre@univ-tlse3.fr (L.T.)

**Keywords:** chronic gamma radiation, microgravity, microgreens, space farming, space exploration, Mars colonization, ionizing radiation

## Abstract

Plants in space face unique challenges, including chronic ionizing radiation and reduced gravity, which affect their growth and functionality. Understanding these impacts is essential to determine the cultivation conditions and protective shielding needs in future space greenhouses. While certain doses of ionizing radiation may enhance crop yield and quality, providing “functional food” rich in bioactive compounds, to support astronaut health, the combined effects of radiation and reduced gravity are still unclear, with potential additive, synergistic, or antagonistic interactions. This paper investigates the combined effect of chronic ionizing radiation and reduced gravity on *Brassica rapa* seed germination and microgreens growth. Four cultivation scenarios were designed: standard Earth conditions, chronic irradiation alone, simulated reduced gravity alone, and a combination of irradiation and reduced gravity. An analysis of the harvested microgreens revealed that growth was moderately reduced under chronic irradiation combined with altered gravity, likely due to oxidative stress, primarily concentrated in the roots. Indeed, an accumulation of reactive oxygen species (ROS) was observed, as well as of polyphenols, likely to counteract oxidative damage and preserve the integrity of essential structures, such as the root stele. These findings represent an important step toward understanding plant acclimation in space to achieve sustainable food production on orbital and planetary platforms.

## 1. Introduction

Higher plants are key components for establishing bioregenerative life support systems (BLSSs), which are crucial for sustaining long-term human missions beyond Earth. These systems are essential for sustaining long-duration space missions, providing astronauts with critical resources such as oxygen, water, and food [1]. Cultivating plant-based fresh food onboard spacecraft, and in situ on the Moon and Mars, within specialized compartments where all environmental factors are carefully controlled can effectively address astronauts’ nutritional needs [2,3]. Moreover, ensuring a balanced diet is crucial for maintaining health and well-being during prolonged space missions. However, the isolation, confinement, and exposure to extreme space conditions, including ionizing radiation and reduced gravity, can lead to various space-induced physiological and psychological issues. Incorporating fresh, plant-based foods rich in bioactive compounds, such as antioxidants, vitamins, minerals, and fibers, can serve as a nutritional countermeasure to prevent and mitigate such risks and contribute positively to astronauts’ psychological well-being [4,5,6].

However, growing plants in space poses significant challenges due to the unique environmental factors, particularly ionizing radiation and reduced gravity. These stressors can influence plant growth, development, and nutritional quality by inducing molecular and cellular changes that affect gene expression, cell proliferation, differentiation, and photosynthesis [1,7,8,9]. The resulting alterations can lead to structural and functional abnormalities, particularly in the early growth stages. For instance, microgravity does not entirely prevent plant reproduction but can negatively affect embryo quality and seedling development [10]. Seeds produced in space often display delayed development, altered storage reserves, and reduced cell numbers in cotyledons [11]. Understanding acclimation and adaptation mechanisms to space factors is critical for ensuring plant growth and reproduction on extraterrestrial surfaces, such as the Moon and Mars, where sustainable seed-to-seed cycles will be necessary for producing food independently of Earth’s resources [12].

Ionizing radiation is a major constraint for space exploration, yet research on plant responses to radiation remains limited compared to other organisms. The interaction between ionizing radiation and biological systems can generate reactive oxygen species (ROS), leading to oxidative stress that damages proteins, lipids, starch, and nucleic acids. Plants have evolved defense mechanisms, both enzymatic and non-enzymatic, to counteract this oxidative damage [13,14]. Notably, plants tend to be more radio-resistant than animals due to differences in their cellular structure and metabolism. While radiation is generally harmful to mammals, it can sometimes have beneficial effects on plants at specific doses. This phenomenon, known as “hormesis” [15], can lead to enhanced stress tolerance, faster germination, and increased production of valuable phytochemicals, such as phenolic compounds, which are important for crops grown for food [1,4,16].

Nevertheless, significant gaps in knowledge remain, particularly regarding the fidelity of Earth-based radiation analog studies. Many experiments have used radiation types and doses that do not accurately reflect the conditions in space [13,17]. While radiation exposure on the International Space Station (ISS) presents acceptable health risks, future missions to the Moon, Mars, and other solar system bodies will subject organisms to chronic exposure from galactic cosmic rays (GCR) and solar particle events (SPE). These exposures are far greater than those encountered on Earth, and resistance to acute high-dose radiation does not necessarily translate into resistance to chronic low-dose radiation, also over multiple generations [18].

Understanding the interplay between different environmental stressors, such as chronic radiation and altered gravity, is crucial for advancing space agriculture. Stress interactions may amplify biological effects, creating complex challenges that may not be evident when stresses are studied in isolation [19]. For example, while Deng et al. [20] demonstrated that simulated microgravity can impair DNA repair mechanisms following acute radiation exposure, the combined impact of chronic radiation and reduced gravity remains poorly understood [19,21,22]. Investigating these interactions is essential, as plants in extraterrestrial environments will simultaneously face multiple stressors. By addressing these gaps, researchers can develop robust strategies to enhance plant tolerance and ensure sustainable agriculture in the extreme conditions of space exploration. Within this framework, the COMBO-AGR project (COMBined effect Of Altered Gravity and chronic Radiation on plants) has been selected by the European Space Agency (ESA) within the Call for Ideas SciSpacE CORA—Ground-Based Facility (GBF) (Ref. I-2023-07518) and achieved admission to the utilization of the test platform MarSimulator, developed by the GSBMS (Scientific group of space biology and medicine, Paul Sabatier University of Toulouse III). The MarSimulator is an innovative device that allows for combining chronic exposure to radiation with alteration of gravity [23]. The central hypothesis of COMBO-AGR is that simultaneous exposure to reduced gravity and chronic ionizing radiation may result in plant responses that differ significantly from those observed under the action of the isolated stressors. The effects of combined stressors may be additive, synergistic, or antagonistic, with various biological targets affected at different levels—from molecular processes to whole-plant metabolism. Understanding whether these two main space factors act in combination or independently is crucial to enable the cultivation of edible plants in space for the upcoming missions to the Moon, Mars, and beyond.

The study reported herein, therefore, focuses on exploring the effects of simulated space conditions on seed germination and microgreen development in *Brassica rapa* L. This fast-growing crop holds significant potential for space agriculture due to its adaptability, high nutritional content, and short cultivation cycle [6]. Indeed, it is considered among candidate species for crop production in space also as “pick and eat” fresh food [24,25]. By investigating how simulated space environments impact its germination and growth, the COMBO-AGR project aims to contribute valuable insights into the development of sustainable and efficient crop production systems to ensure a reliable, nutrient-dense food source to complement astronauts’ diets during extended missions.

Specifically, we examined the effects of chronic low-dose gamma radiation, mimicking the levels expected during a Mars mission (0.66 mSv/d), and reduced gravity on seedling growth and development, with a focus on the signs of oxidative stress through microscopy techniques. The cultivation cycle was stopped at the microgreens stage for the volume limitations imposed by the facility used to simulate the space factors. To test our hypotheses, we designed an experiment with four treatments: (1) control: plants grown under standard Earth conditions; (2) chronic irradiation: plants exposed solely to chronic doses of gamma rays; (3) reduced gravity: plants subjected only to simulated reduced gravity; and (4) an interaction of factors: plants exposed simultaneously to chronic radiation and reduced gravity.

By analyzing the responses of *B. rapa* microgreens to these treatments, we aimed to elucidate the potential challenges and opportunities for cultivating plants in space environments with the goal of developing strategies for sustainable food production during long-term space missions and future Mars colonization efforts.

## 2. Results

### 2.1. Morphological and Biometric Analyses

In all the morphological parameters analyzed (Table 1), the effect of gravity as a main factor was not significant, while the effect of IR was significant for fresh weight and the hypocotyl/root ratio. Specifically, the exposure to chronic gamma radiation determined a decrease in the microgreens’ fresh weight and an increase in the hypocotyl/root ratio compared to not-irradiated ones.

**Figure 1:**
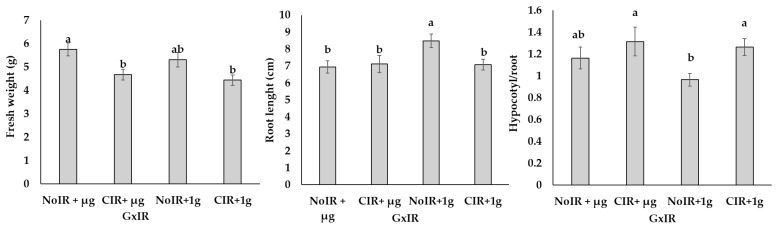
Effect of the interaction between the main factors (G and IR) on fresh weight, root length, and hypocotyl/root ratio of microgreens of *B. rapa*. Mean values and standard errors are shown. Different letters correspond to significant differences among irradiation doses according to the Student–Newman–Keuls multiple comparison tests (*p*-value ≤ 0.05).

Furthermore, the interaction between the main factors (G × IR) was significant for fresh weight, root length, and the hypocotyl/root ratio (Figure 1). In particular, the fresh weight was significantly lower in irradiated conditions, both at 1 g and µg, compared to the seedlings grown under µg conditions without additional irradiation. Additionally, NoIR + 1 g seedlings displayed significantly longer roots compared to seedlings developed in all the other conditions. As regards the hypocotyl/root ratio, the seedlings developed under irradiated conditions showed significantly higher values than the NoIR + 1 g seedlings.

Regarding the coefficient of variation of the aforementioned parameters (Table 2), the CIR + µg treatment showed the highest variability for hypocotyl (16.6%) and root length (30.1%) and the hypocotyl/root ratio (42.2%). In contrast, variability was generally lower under 1 g conditions (NoIR + 1 g and CIR + 1 g). The cotyledon area exhibited relatively consistent variability across all treatments, with the highest values observed for NoIR + µg and CIR + µg (32.5%).

### 2.2. Anatomical Analyses

**Figure 2:**
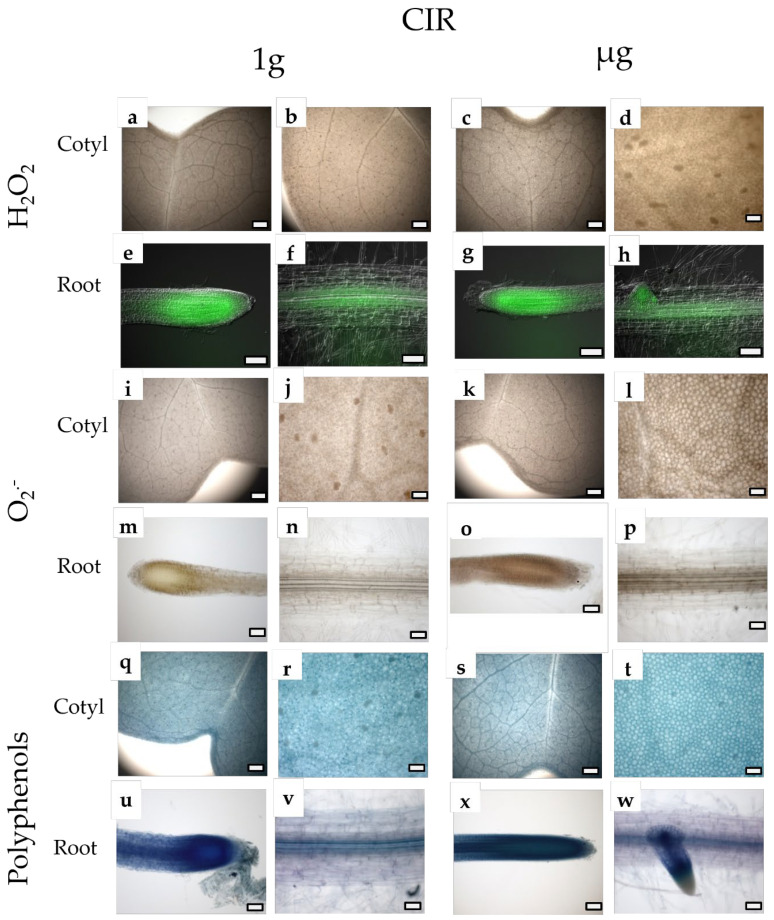
Light and epifluorenscence microscopy views of *B.rapa* L. cotyledons and roots from irradiated samples (CIR) in presence of µg (CIR + µg; (**c**,**d**,**g**,**h**,**k,l**,**o**,**p**,**s**,**t**,**w**,**x**)) and in presence of 1 g (CIR + 1 g; (**a**,**b**,**e**,**f**,**i**,**j**,**m**,**n**,**q**,**r**,**u**,**v**)) and stained with DAB (**a**–**d**), FDA (**e**–**h**) NTB (**i**–**p**) and TBO (**q**–**x**). Images (**a**–**c**,**i**,**k**,**l**,**q**–**t**) are all at the same magnification; bar = 200 μm. Images (**d**,**j**,**k**,**m**–**p**,**u**–**x**) are all at the same magnification; bar = 50 μm. Images (**e**–**h**) are all at the same magnification; bar = 100 μm.

**Figure 3:**
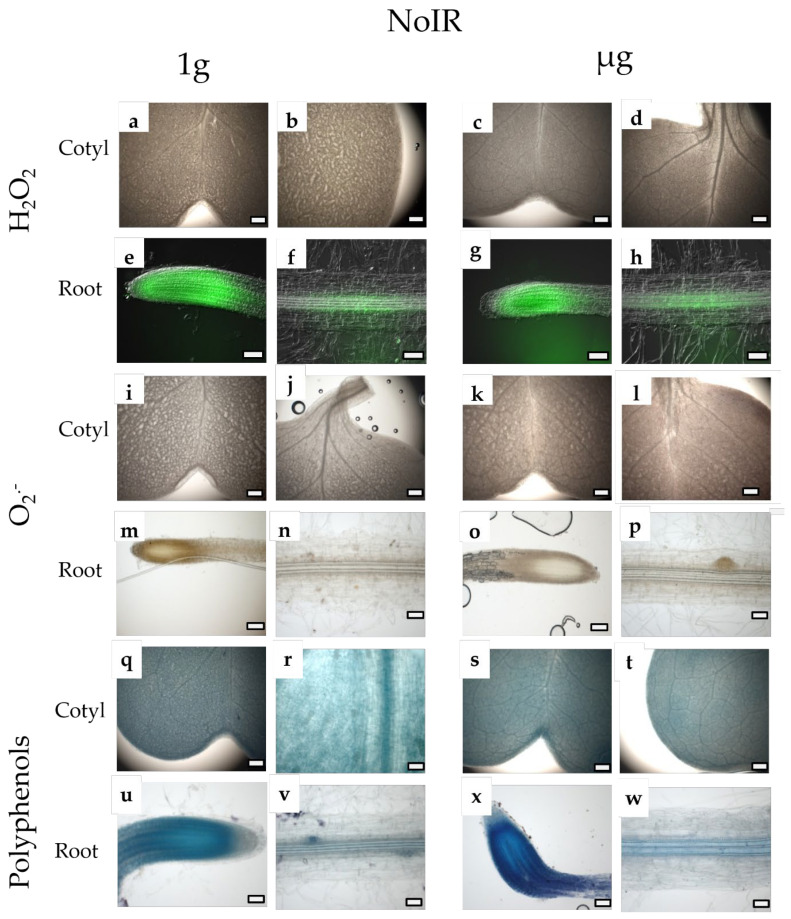
Light and epifluorenscence microscopy views of *B.rapa* L. cotyledons and roots from not irradiated samples (NoIR), in presence of µg (NoIR + µg; (**c**,**d**,**g**,**h**,**k**,**l**,**o**,**p**,**s**,**t**,**w**,**x**)) and in presence of 1g (NoIR + 1 g; (**a**,**b**,**e**,**f**,**i**,**j**,**m**,**n**,**q**,**r**,**u**,**v**)) and stained with DAB (**a**–**d**), FDA (**e**–**h**) NTB (**i**–**p**) and TBO (**q**–**x**). Images (**a**–**d**,**i**–**l**,**s**,**t**) are all at the same magnification; bar = 200 μm. Images (**e**–**h**,**m**–**p**) are all at the same magnification; bar = 100 μm. Images (**q**,**r**,**u**–**x**) are all at the same magnification; bar = 50 μm.

Microscopy analyses (Figure 2 and Figure 3) showed that neither the gravity level nor irradiation influenced the localization of H_2_O_2_ and O_2_^−^ in both the cotyledons and root apexes. The distribution of polyphenols in the roots was different in seedlings grown under the combination of CIR and simulated µg, with the whole stele being homogeneously stained, compared to the other treatments, where the dyes were localized in the middle and at the periphery of the stele. Irradiation determined a general increase in the intensity of staining with NBT and toluidine blue in roots when developed in simulated µg, suggesting an increase in the accumulation of O_2_^−^ and polyphenols. Indeed, in the roots subjected to the combined stress of radiation and microgravity (Figure 2w,x), a significantly enhanced and distinct accumulation of polyphenols was observed, particularly within the central cylinder (stele) and the root meristematic zone, compared to the control roots grown under 1 g conditions and without radiation exposure (Figure 3u,v). A slight increase in the intensity of polyphenol staining was also observed in irradiated seedlings under 1 g conditions compared to non-irradiated ones.

## 3. Discussion

This study investigated the individual and combined effects of chronic gamma radiation and simulated reduced gravity on *Brassica rapa* L. microgreens, suggesting a good tolerance towards both the two main stressors, as well as their interaction. The germination percent was not affected by the treatments, although deeper analyses suggested the occurrence of signs of oxidative stress in response to these two space-relevant environmental factors, with possible implications for plant growth and development at further stages of development.

While altered gravity alone did not significantly influence the morphological parameters measured, chronic radiation exposure had a marked effect on fresh weight and the hypocotyl/root ratio. This suggests that *B. rapa* microgreens may be more sensitive to radiation stress than to altered gravity conditions during the early growth stages. Indeed, the hypocotyl and root length are the result of cell proliferation and expansion processes, which are reported to be altered in real or simulated microgravity, thus resulting in a variety of responses, including increased, decreased, or unchanged axis length [26]. Previous studies on *Arabidopsis thaliana* (L.) Heynh. have shown that, in microgravity, roots often exhibit increased elongation and a disoriented growth pattern, such as bending and skewing, likely due to disrupted gravitropic responses [27,28]. This skewing effect is further compounded by the relocalization of auxin efflux carriers, which are critical for root growth directionality [29]. The altered auxin distribution in microgravity leads to changes in root meristematic cell dynamics, resulting in increased proliferation rates in some cases, while in others, it may lead to reduced root growth due to the lack of directional cues [30,31]. Other studies demonstrate that exposure to low-LET ionizing radiation induces variability in morpho-physiological traits, as shown in *Eruca sativa* Mill., where exposure to 1 Gy increased the shoot length and leaf number, while a 10 Gy dose led to longer roots [32]. However, chronic low-dose exposure, such as 35 μGy/h, did not significantly impact root growth or overall morphology across generations of *A. thaliana*. In other studies, *E. sativa* and *Solanum lycopersicum* L. seeds exposed to cosmic radiation on the ISS and then exposed to similar doses of Cs-137 did not show changes in growth or root length in either species, suggesting tolerance in certain species [33,34]. Interestingly, in another study, *Vicia faba* L., *Vigna radiata* L., and *Pisum sativum* L. plants exposed to low levels of gamma radiation exhibited compensatory growth, with improved root and shoot lengths compared to controls, suggesting possible adaptive mechanisms in response to mild stress [35].

The decrease in fresh weight observed in our irradiated plants, regardless of gravity conditions, indicates that chronic low-dose radiation in *B. rapa* may impair overall biomass accumulation, potentially due to radiation-induced oxidative stress or alterations in metabolic processes. The lower hypocotyl/root ratio in non-irradiated plants further suggests that radiation exposure may also alter the resource allocation between above- and below-ground tissues. Interestingly, the interaction between gravity and radiation produced significant effects on fresh weight, root length, and the hypocotyl/root ratio. This highlights the importance of considering these factors in combination, as their joint impact may not be predictable from their individual effects. For instance, the observation that the 1 g plants exhibited the longest root length under non-irradiated conditions suggests that normal gravity promotes root growth, but the combined stress of radiation and microgravity nullifies this effect. The alteration of root and hypocotyl length due to space factors may pose a critical challenge for sustainable plant cultivation systems also in space since nutrient uptake, structural integrity, and anchorage are essential. Therefore, research is active in this field also to find technical solutions to modulate and orient root growth by controlling other environmental and cultivation factors [26,36]. The observed phenomena suggest that the combination of irradiation and simulated microgravity induces a synergistic stress response in plants, particularly in terms of oxidative stress and polyphenol accumulation. Indeed, both factors are recognized to induce the production of reactive oxygen species (ROS) [13,37], such as hydroxyl radicals, singlet oxygen, and hydrogen peroxide, and activate signaling pathways that influence physiological, biochemical, and molecular mechanisms. However, excessive ROS leads to oxidative stress, causing damage to lipids, nucleic acids, proteins, and metabolites, which can ultimately result in cell death [38]. In response, the plants rely on enzymatic antioxidant systems (superoxide dismutase, catalase, and peroxidases) and non-enzymatic antioxidants (polyphenols, flavonoids, and tocopherols), which neutralize ROS and protect cellular structures, increasing the production of polyphenols, secondary metabolites with antioxidant properties that help mitigate the oxidative damage [14,39,40,41,42]. Simultaneously, plants activate DNA repair mechanisms, such as homologous recombination and excision repair systems, to preserve genomic integrity.

Our experimental results align with these mechanisms, revealing increased polyphenol accumulation in key root zones, particularly the central cylinder (stele) and the meristematic region, especially under the combined stress of radiation and microgravity (CIR + µg), indicating a systemic defense response. This specific distribution pattern highlights a critical adaptive response, whereby polyphenols are concentrated in key functional regions of the root to mitigate the detrimental effects of the combined stressors. The central cylinder, housing the vascular tissues (xylem and phloem), is vital for nutrient and water transport, while the meristematic zone represents the primary site of cell division and growth, rendering these areas particularly vulnerable to oxidative damage [43]. This spatial accumulation of polyphenols likely reflects a protective mechanism to preserve the structural and functional integrity of these critical root regions under environmental stress. Our observations align with the broader mechanisms of oxidative stress responses, highlighting the critical role of polyphenols. These compounds function as powerful antioxidants and structural stabilizers, significantly contributing to plant tolerance against abiotic stresses, including salinity, drought, extreme temperatures, heavy metals, and UV radiation [44]. For instance, Chen et al. [45] demonstrated that polyphenol content is significantly higher in aluminum-tolerant lettuce genotypes. This increased accumulation was linked to an adaptive strategy to protect the plant from oxidative stress. Moreover, the roots are the organs where they accumulate most extensively. For example, AbdElgawad et al. [46] reported a significant increase in polyphenol levels in maize roots under salinity stress. The accumulated polyphenols play a crucial role in neutralizing the ROS generated by salinity and may also have signaling functions in the roots, enabling the activation of systemic defense mechanisms in the aerial parts of the plant. In light of our results, the observed accumulation of polyphenols in the root stele and meristematic zone under combined radiation and microgravity stress reflects a similar protective and signaling role.

Radiation and microgravity, either individually or in combination, have been observed to significantly inhibit root growth. Xu et al. [47] established a strong connection between gallic acid, a polyphenolic compound, and the regulation of root growth. Gallic acid inhibits root growth by reducing the size of the root meristem and blocking cell division in this region. It also significantly decreases auxin (IAA) levels in root apices by inhibiting the expression of key auxin efflux transporters (PIN1, PIN2, PIN3, and PIN7), which are essential for polar auxin transport. As an antioxidant polyphenol, gallic acid influences ROS levels and hormonal signaling, establishing a link between secondary metabolites and plant hormones. This inhibition of growth can be interpreted as an adaptive strategy that allows the plant to conserve metabolic resources for sustaining long-term stress responses. Polyphenols, as specialized secondary metabolites, demonstrate remarkable plasticity in their responses to abiotic stresses. Their role extends beyond simple ROS neutralization, encompassing structural, signaling, and acclimation functions that are critical for plant tolerance in hostile environments.

## 4. Conclusions

This study revealed insights into the tolerance responses of *Brassica rapa* L. microgreens exposed to the combined effects of chronic gamma radiation and simulated reduced gravity. Germination remained unaffected by the treatments, but the radiation stress significantly influenced the morphological traits, such as fresh weight and the hypocotyl/root ratio, whereas gravity alone showed negligible effects. Notably, the interaction of radiation and microgravity produced synergistic stress responses, underscoring the importance of studying these factors together rather than in isolation.

The findings emphasize the critical role of polyphenols in detoxifying ROS, stabilizing root structures, and mediating signaling pathways under combined stressors. By demonstrating localized polyphenol accumulation in the stele and meristematic regions, this work reveals how plants manage resource allocation and prioritize defense mechanisms in key functional zones under multi-stressor conditions. The observed inhibition of root growth, especially under combined stress conditions, may represent an acclimation strategy, allowing plants to conserve metabolic resources while prioritizing defense mechanisms.

Our findings extend the understanding of plant responses to space-relevant stressors, emphasizing the resistance of *B. rapa* microgreens and the intricate roles of secondary metabolites in plant adaptation. The results can have significant implications for sustainable agriculture in extraterrestrial environments, where optimizing root function and biomass production are critical for nutrient acquisition and plant stability. Future research should focus also beyond the microgreens stage on unraveling the molecular pathways involved in ROS detoxification, particularly the interplay between enzymatic antioxidants and polyphenol biosynthesis to enhance resistance in space-grown crops.

## 5. Materials and Methods

### 5.1. Experimental Design

The study employed a 2 × 2 factorial design to investigate the individual and combined effects of ionizing radiation (IR) and gravity (G) on seed germination and microgreen development in *Brassica rapa* L. The microgreens stage was selected primarily due to the volume constraints of the random position machine (RPM; Dutch Space, Leiden, The Netherlands), which restricts the volume available for experimental samples. Microgreens, being smaller than mature plants, are better suited for the efficient use of this limited space while maintaining the integrity of the experimental design. Additionally, microgreens are highly valued for their nutritional content, as they are rich in bioactive compounds, such as vitamins, antioxidants, and minerals, making them an important “pick-and-eat” food that is easily produced onboard. For IR, two levels were applied: non-irradiated control (NoIR) with no additional radiation exposure (0 mSv/day) and chronic gamma radiation exposure treatment (CIR) set at 0.66 mSv/day, corresponding to low-dose chronic exposure expected in the Mars environment [48]. For G, two levels were set: the 1 g control (1 g) at standard Earth gravity and a reduced gravity level simulating microgravity (µg).

**Figure 4:**
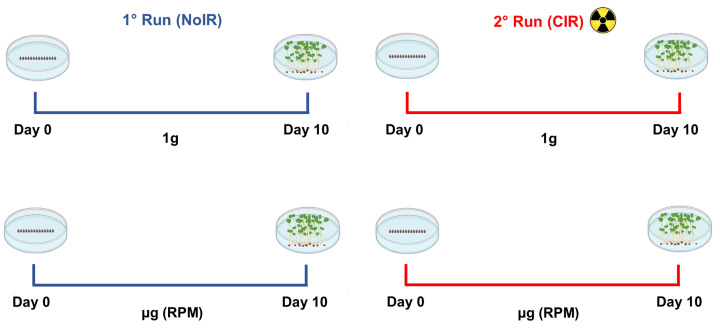
Schematic view of the experimental design of the study.

This factorial design (Figure 4) yielded four distinct experimental conditions. The first was the control condition (NoIR + 1 g). The second condition involved radiation only (CIR + 1 g). The third condition focused solely on reduced gravity (NoIR + µg). The last condition was the combined treatment condition (CIR + µg).

### 5.2. Implementation of the Trial

The experiment was conducted at the MarSimulator facility, part of the Space Biology and Medicine Scientific Group (GSBMS) at Paul Sabatier University of Toulouse III (France).

**Figure 5:**
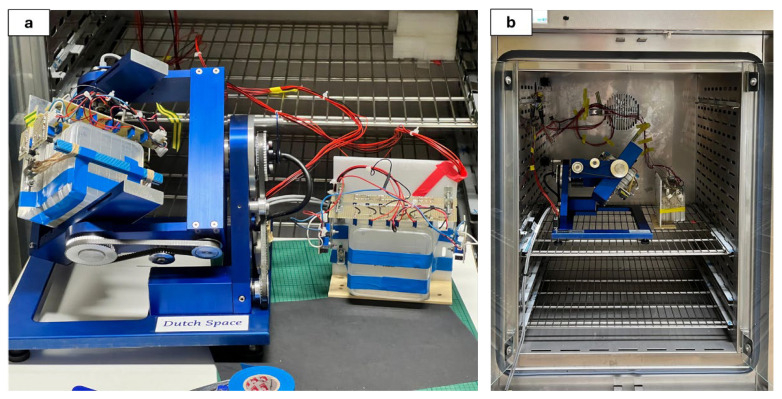
Example of sample configuration in the MarSimulator device for NoIR + 1 g and NoIR + µg conditions. (**a**) displays Petri dishes mounted on the rpm (NoIR + µg) and on the vertical support (NoIR + 1 g). (**b**) shows the samples inside the phytotron.

Dry seeds of *Brassica rapa* L. subsp. *sylvestris* var. *esculenta* (Bioseme s.c.a.r.l., Piombino, Italy) were disinfected by sequentially rinsing them in 95% ethanol, followed by a 1% calcium hypochlorite solution and then rinsed with sterile water, with each step lasting 5 min. After disinfection, 60 seeds were incubated in petri dishes equipped with 40 mL of sterilized half-strength Murashige–Skoog medium. For each treatment, 4 Petri dishes, each with 60 seeds, were used. These dishes were incubated in the MarSimulator device (Figure 5), which consists of a cell incubator equipped with a RPM, as well as a thorium-based radiation source. The choice of 4 replicates was dictated by the volume constraints of the RPM. Despite this limitation, the use of 4 replicates ensured sufficient statistical reliability to detect significant biological effects while maintaining feasibility within the experimental design. For the NoIR + µg and CIR + µg treatments, the Petri dishes were mounted on the RPM, while for the NoIR + 1 g and CIR + 1 g treatments, they were positioned upright on a vertical support (Figure 5). Incubation lasted for 10 days under controlled environmental conditions, with a temperature of 22 °C, a light intensity of 40 µmol/m^2^/s, and a 16 h photoperiod. The phytotron provided precise temperature regulation, while the LEDs were calibrated with a photometer to ensure uniform and consistent light intensity throughout the experiment.

To monitor radiation exposure (CIR), two passive dosimeters (RPL) were placed on top of the control Petri dishes (CIR + 1 g) and two more on the dishes mounted on the RPM (CIR + µg).

#### 5.2.1. Radiation Treatment

According to projections, a 652-day mission to Mars during the solar activity minimum is expected to result in a cumulative radiation dose ranging from 0.6 Sv (the maximum career dose tolerated by NASA) to 1 Sv (the maximum recommended dose by the ICRP) [48,49,50,51]. Estimating radiation doses is a critical step in mission planning, enabling the optimization of protective measures during long-duration space exploration. To simulate chronic gamma radiation exposure, thorium nitrate was used as the radiation source. The thorium nitrate powder was sealed within three layers of airtight plastic bags (33 × 36.5 cm^2^) with a total thickness of 0.4 to 1.2 cm, enclosed in a protective cardboard envelope (32 × 44 cm^2^), and covered with an additional airtight plastic bag to create a sealed source suitable for placement in a cell incubator. The primary isotope, ^232^Th, emits alpha radiation with 4 MeV energy and gamma radiation with 60 keV energy [17], with the radiation dose rate estimated at 0.66 mSv per day. Indeed, alpha particles emitted by ^232^Th are easily stopped by the protective cardboard envelope, which is instead crossed by gamma radiation. This situation mimics the conditions on the ISS or future space travel to the Moon or Mars, where the majority of the radiation capable of passing through the protective shielding layers consists of gamma rays.

#### 5.2.2. Reduced Gravity Simulation

Reduced gravity conditions were simulated using a Random Positioning Machine (RPM). The RPM was positioned directly on top of the radiation source when performing the trial for simultaneous exposure to both reduced gravity and chronic radiation [17].

The parameters used for this simulation were random interval, speed, and direction. The speed was framed to 6 to 60 rpm.

### 5.3. Morphological and Biometric Traits

Ten days after sowing, the seedlings were carefully removed from the medium and the percentage of germination and fresh weight were determined. For 30 seedlings per petri dish, the following data were recorded: hypocotyl and root length, cotyledon area, and the hypocotyl/root ratio. For the root length measurements, only roots with an intact apex were considered. The remaining seedlings were fixed in FAA (formaldehyde 38%/glacial acetic acid/ethanol 50%, 5/5/90 in volume) for subsequent microscopy analyses.

### 5.4. Microscopy Analyses

The fixed seedlings were dissected into cotyledons and root apex and were subjected to different staining procedures to highlight hydrogen peroxide (H_2_O_2_), superoxide ion (O_2_^−^), and polyphenols in at least 3 replicates per treatment. According to Daudi et al. [52], labeling of H_2_O_2_ was carried out by submerging the cotyledons in a 3,3′-Diaminobenzidine (DAB-HCl) (Sigma-Aldrich, St. Louis, MO, USA) solution at 1 mg/mL. The labeling was carried out for 12 h at room temperature, in darkness, with gentle agitation (40 rpm). The cotyledons were then cleared of chlorophyll by immersing them in 95% ethanol for 10 min at 70 °C, followed by 30 min at room temperature. They were subsequently observed under a Zeiss (Carl-Zeiss, Goettingen, Germany) Imager M2 microscope in bright-field mode. Labeling for H_2_O_2_ in root apexes was performed through 2′,7′-Dichlorofluorescein Diacetate (H2DCFDA), according to Ben Rejeb et al. [53]. In brief, the roots were immersed in a 25 µM H2DCFDA (Sigma-D6883) solution in 20 mM potassium phosphate buffer at pH 6. After 15 min of incubation at room temperature in darkness, the roots were observed under a Zeiss Imager M2 fluorescence microscope equipped with the ApoTome system and GFP filter (excitation: 530 nm/emission: 504 to 525 nm). For the detection of O_2_^−^, labeling with Nitro Blue Tetrazolium (NBT) was applied according to Tyburski et al. [54]. Specifically, the cotyledons and roots were incubated for 30 min and 10 min, respectively, at room temperature in an NBT (Sigma-N6639) solution at 0.05 mg/mL in 0.1 M phosphate buffer at pH 7.4. The cotyledons were then decolorized in 95% ethanol at 70 °C for 10 min, followed by 30 min at room temperature. The cotyledons and roots were observed under a Zeiss Imager 2 microscope. Finally, the labeling of polyphenols was conducted with Toluidine Blue according to Ribeiro and Leitao [55]. Before polyphenol labeling, the cotyledons were pre-decolorized with 95% ethanol for 10 min at 70 °C, followed by 30 min at room temperature. The cotyledons and roots were then immersed for 30 s and 10 s, respectively, in a 0.01% TBO (Sigma-89640) solution in McIlvain buffer at pH 4. The cotyledons and roots were observed under a Zeiss Imager 2 microscope.

### 5.5. Statistical Analysis

All data were statistically analyzed using SPSS^®^ software (Statistics for Data Analysis V. 29, SPSS Inc., Chicago, IL, USA). The Shapiro–Wilk and Kolmogorov–Smirnov tests were conducted for the preliminary assessment of normality. Additionally, a preliminary transformation using the arcsine function was applied to the percentage data. A two-way ANOVA was then performed, followed by the Student–Newman–Keuls post hoc test with a significance level set at *p* < 0.05, considering the two levels of radiation (IR—NoIR and CIR) and gravity (G—1 g and µg) as the main factors, as well as their interaction (G × IR). The coefficient of variation was calculated as the standard deviation divided by the mean value and was calculated for the germination and biometric parameters.

## Figures and Tables

**Table 1:** Effect of the different levels of gravity (G) and radiation (IR) as main factors and of their interaction (G × IR) on germination percentage, fresh weight, hypocotyl length, root length, and cotyledons area of microgreens of *Brassica rapa* L. Mean values, standard errors, and significance levels are shown. Different letters correspond to significant differences among irradiation doses according to the Student–Newman–Keuls multiple comparison tests (*p*-value ≤ 0.05). NS: not significant; *, **: significant at *p*-value < 0.05 and 0.01, respectively.

	Germination (%)	Fresh Weight (g)	Hypocotyl Lenght (cm)	Root Lenght (cm)	Cotyledons Area (cm^2^)	Hypocotyl/Root
**Gravity (G)**						
1g	99 ± 0.83 a	4.88 ± 0.243 a	8.235 ± 0.168 a	7.728 ± 0.271 a	0.173 ± 0.005 a	1.125 ± 0.055 a
µg	100 ± 0 a	5.218 ± 0.262 a	8.154 ± 0.182 a	7.046 ± 0.325 a	0.183 ± 0.006 a	1.251 ± 0.086 a
**Radiation (IR)**						
NoIR	100 ± 0 a	5.539 ± 0.21 a	8.047 ± 0.176 a	7.842 ± 0.306 a	0.178 ± 0.006 a	1.047 ± 0.055 b
CIR	99 ± 0.83 a	4.559 ± 0.153 b	8.332 ± 0.172 a	7.095 ± 0.283 a	0.178 ± 0.005 a	1.287 ± 0.073 a
**Significance**						
**G**	NS	NS	NS	NS	NS	NS
**IR**	NS	**	NS	NS	NS	*
**G × IR**	NS	*	NS	*	NS	*

**Table 2:** Coefficient of variation of germination, fresh weight, hypocotyl length, root length, cotyledon area, and hypocotyl/root ratio of *B. rapa* microgreens from: irradiated samples (CIR) in presence of µg (CIR + µg) and in presence of 1 g (CIR + 1 g); not irradiated samples (NoIR) in presence of µg (NoIR + µg) and in presence of 1 g (NoIR + 1 g).

	Coefficient of Variation (%)					
	Germination	Fresh Weight	Hypocotyl Lenght	Root Lenght	Cotyledons Area	Hypocotyl/Root
**NoIR + µg**	0.00	9.56	16.4	18.7	32.5	30.6
**CIR + µg**	0.00	9.63	16.6	30.1	32.5	42.2
**NoIR + 1g**	3.22	11.8	15.3	20.0	30.7	25.4
**CIR + 1g**	0.00	10.0	14.2	20.1	31.6	28.3

## Data Availability

The data that support the findings of this study are available from the corresponding authors upon reasonable request.
